# Stereological analysis of elastic fibers of the corpus cavernosum of rats during the aging process^1^

**DOI:** 10.1590/s0102-865020190080000003

**Published:** 2019-10-14

**Authors:** Thiago Hota, Fernando Lorenzini, Eduardo Felippe Melchioretto, Marcelo Zeni, Djanira Aparecida da Luz Veronez, Rogério de Fraga

**Affiliations:** I Fellow Master degree, Postgraduate Program in General Surgery, Universidade Federal do Paraná (UFPR), Curitiba-PR, Brazil. Design, intellectual and scientific content of the study; acquisition and interpretation of data; manuscript preparation and writing.; II PhD, Department of Urology, School of Medicine, UFPR, Curitiba-PR, Brazil. Conception of the study, manuscript writing, critical revision.; III Fellow Master degree, Postgraduate Program in General Surgery, UFPR, Curitiba-PR, Brazil. Acquisition of data.; IV PhD, Professor, Department of Anatomy, Medical School, UFPR, Curitiba-PR, Brazil. Design, intellectual and scientific content of the study.; V PhD, Associate Professor, Department of Urology, Medical School, UFPR, Curitiba-PR, Brazil. Design, intellectual and scientific content of the study.

**Keywords:** Elastic Tissue, Erectile Dysfunction, Aging, Rats

## Abstract

**Purpose:**

To evaluate changes in the quantity of elastic fibers in the corpora cavernosa of rats during the natural aging process, and to assess the degree of this change by determining volumetric density (Vv) at different ages via stereological analysis.

**Methods:**

Forty-eight rats, raised under similar conditions, were subjected to the natural aging process and divided into four groups (G1 to G4), according to age at the time of penectomy (6, 9, 12, and 24 months, respectively). Histological sections of the middle segment of the penis were stained with Weigert’s resorcin-fuchsin, and the volumetric density (Vv) of elastic fibers of the corpora cavernosa were determined via stereological analysis.

**Results:**

There were no statistically significant differences in Vv among groups G1, G2, and G3. These three groups were therefore considered as a single group. The mean Vv of this group showed a statistically significant reduction compared to that of G4 (0.16 *vs.* 0.11, p<0.05).

**Conclusion:**

Natural aging in rats was responsible for a reduction in volumetric density of elastic fibers of the corpora cavernosa (approximately 30% decrease in Vv) during senescence.

## Introduction

Erectile dysfunction (ED) is defined as the inability to initiate, maintain, and conclude a satisfactory sexual relationship. According to the Diagnostic and Statistical Manual of Mental Disorders (DSM)-5, symptoms must last for a minimum time of 6 months for diagnosis of ED^1^ . The incidence of ED is high, affecting approximately 152 million men worldwide^2^ .

ED may be caused by one or more comorbidities, and prevalence increases with aging. In addition to the arterial, hormonal, neurological, and veno-occlusive origins of ED, increasing evidence implicates the structural disorganization of corpus cavernosum trabeculae and changes in the content of the fibers that make up the tunica albuginea^2^ .

Trabeculae are composed of endothelial and smooth muscle cells, as well as an extracellular matrix formed by collagen and elastic fibers. The elastic fibers are composed of fibrillar glycoprotein and fibril groups, which occupy extracellular spaces where they are embedded in elastin. This system allows the lengthening and increase of penile stiffness during erection, as well as the rapid return to a flaccid state after detumescence^3^ . As these elastic fibers are produced in the early stages of life, in adulthood, the damaged fibers are repaired ineffectively, with protein material prepared in a disorganized manner and, therefore, may have impaired functional activity^4^ .

The number of elastic fibers is reduced in men with ED compared to those in control subjects of the same age^3^ , allowing us to infer its importance in the erection process. A reduction of these fibers in the corpora cavernosa of rabbits subjected to induced diabetes mellitus was previously reported^5^ , and in another study conducted on healthy rats, a reduction in the volumetric density (Vv) of elastic fibers of the corpora cavernosa was observed in the early stages of adult life^6^ . However, further studies are required to confirm these results^6^ .

Experimental studies, especially those using rats, are a viable and economical option to demonstrate specific changes as rats have a short life span and therefore quickly reach the senescence phase^6^ .

This study aimed to evaluate changes in the number of elastic fibers in the corpora cavernosa of rats during the natural aging process, and to assess the degree of this change by determining Vv at different ages via stereological analysis.

## Methods

This study followed the ethical principles of animal experimentation established by the Brazilian College of Animal Experimentation (COBEA) and the norms of the Canadian Council on Animal Care (1993). It was previously approved by the Ethics Committee on Animal Experimentation (CEEA), of the Sector of Biological Sciences of the Universidade Federal do Paraná, as part of the project “Anatomical and physiological evaluation of male urogenital aging,” process No. 23075.032620/2010-10.

Forty-eight Wistar rats ( *Rattus norvegicus* var *. albinus* ), raised under similar conditions, none of them mate females during their lifetime and were randomly divided into four groups of 12 rats each (G1, G2, G3, and G4). Animals were subjected to resection of the penis and other organs for other research at the ages of 6, 9, 12, and 24 months, respectively. For the surgical procedures, the rats were anesthetized intraperitoneally with a solution of Ketamin Hydrocloride (57.67mg/ml) associated to 2% xylazine hydrochloride at a dose of 1ml/Kg body weight. A one-punch cardiac puncture was carried out and induction of cardiac arrest occurred by exsanguination. This was followed by resection of penis, kidneys, bladder, liver, brain, heart and aorta.

### Histological processing

**Figure 1:**
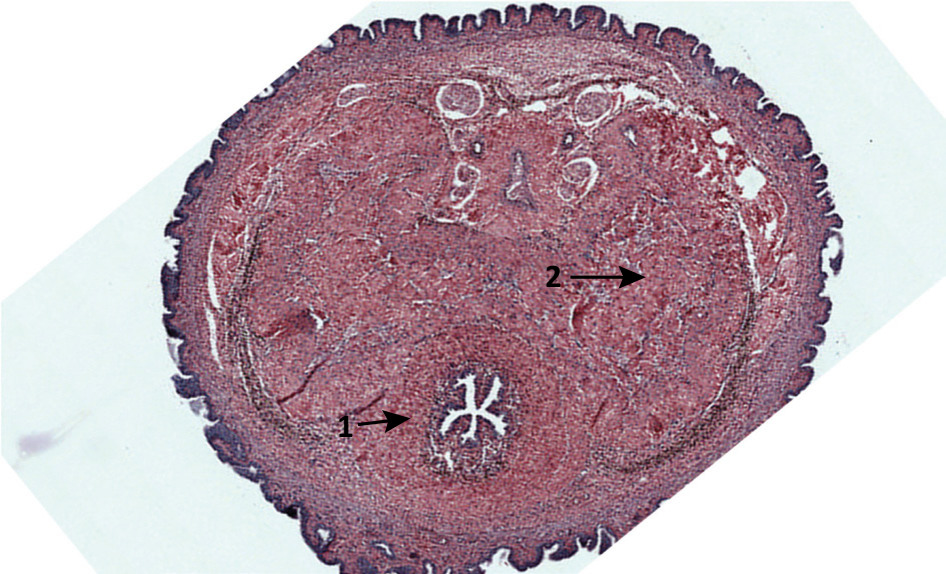
– Penile corpus stained with Weigert’s resorcin-fuchsin. *Arrow 1* , urethra; *arrow 2* , corpus cavernosum. Image was taken from rat number 10 (G1, euthanized at six months).

The penis of each rat was laid on a horizontal surface, and the middle third segment was resected, according to a technique previously described^5^ . After tissue fixation in 10% formalin solution, the segment was kept in fixative solution for 16 hours, and then dehydrated by passage through solutions of xylene and alcohol. Finally, the sections were embedded in paraffin and cut into 5-μm sections using a microtome (American Optical, Spencer AO 820). According to that described by Baddeley *et al.* ^7^ , we used the vertical section method, with isotropic, uniform, and random sections obtained according to the cylindrical shape of the corpora cavernosa and their isotopic compositions. Ten consecutive sections were mounted on slides and stained using Weigert’s resorcin-fuchsin method ( Fig. 1 ). Following the method described by Pinheiro *et al* .^8^ , we did not perform oxidation with Oxone, as there were no differences in staining patterns between the slides treated and not treated with oxidation.

Images of the histological slides were captured using a video camera (Olympus Corporation, Tokyo, Japan) coupled to an optical microscope at x400 magnification and were scanned and analyzed using the VSVviewer^®^ software. In order to increase the accuracy of the stereological analyses, five consecutive sections were evaluated, with five randomly selected fields of the corpora cavernosa analyzed per section (i.e., 25 fields per each rat, totaling 1200 fields).

**Figure 2:**
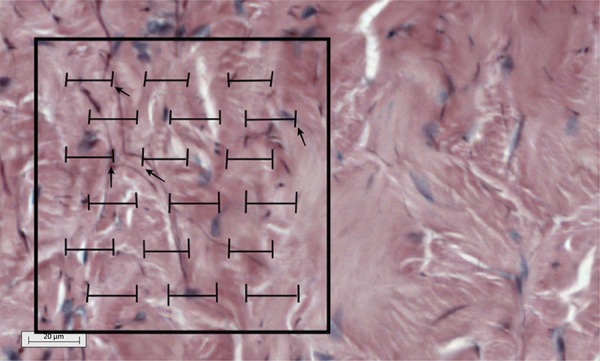
Grid M42 superimposed on the corpus cavernosum. Elastic fibers that contact the test points ( *black arrows* ) were counted. Image taken from rat number 10 (G3, euthanized at 12 months).

Analysis of the points of interest of the images was based on the determination of the Vv of elastic fibers using the grid M42^9^ , comprising 42 test points that were superimposed on the selected image ( Fig. 2 ). Manual counting was performed, without using computerized analysis. The number of test points contacted by elastic fibers was divided by the total number of test points in the field, and the resulting percentage of elastic fibers was used to determine Vv.

Vv was determined according to the following formula: Vv = Pt/Pp, where Pt = number of total points (test), and Pp = number of partial points (number of points contacted by fibers – number of positive points).

### Statistical analysis

A Pearson correlation statistical test was used to evaluate the association between the ages of rats and their respective elastic fiber Vv. Welch’s *t* -test was used to assess the statistical significance of the differences in Vv values observed between groups. Statistical analyses were performed using the R software. A significance level of 95% was used (p<0.05).

## Results

**Figure 3:**
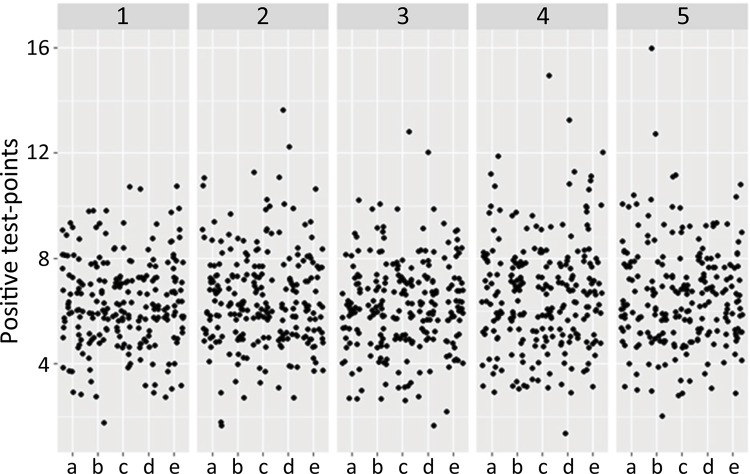
- Random fields of corpus cavernosum (a-e) in random sections (1-5).

Histological section fields were found to be homogeneous. The positive points of the field test, which are those contacted by elastic fibers in each random vertical section of the corpus cavernosum, are shown in Figure 3 numbered from 1 to 5. Five fields were analyzed and labeled “a” to “e”.

**Figure 4:**
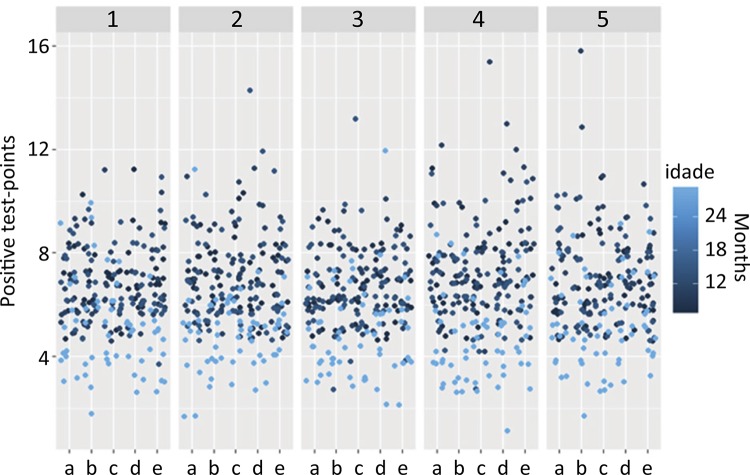
Random fields of corpus cavernosum (a-e) in random sections (1-5). Showing the homogeneity corresponding to the age of the rats.

The homogeneity of positive test points, corresponding to the age of the rats, is shown in ( Fig. 4 ).

**Figure 5:**
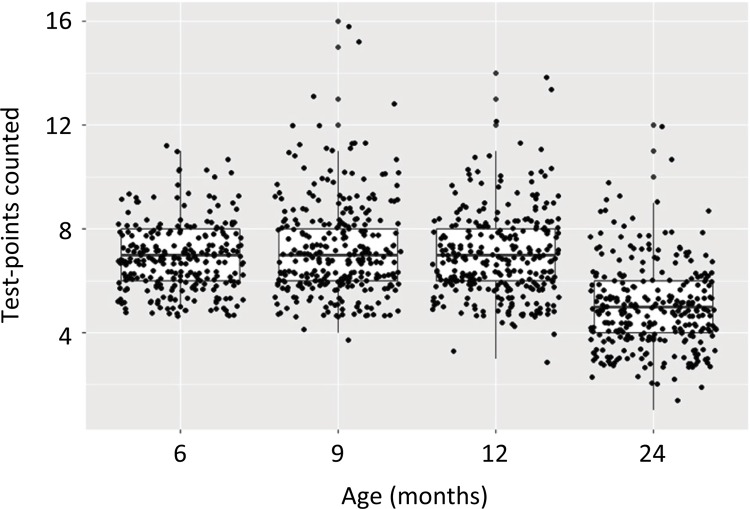
Variation of positive test-points in different ages.

Positive test points (those contacted by elastic fibers) are shown in the digitalized images of the rats’ corpora cavernosa (G1 to G4), grouped according to age ( Fig. 5 ).

The Pearson correlation coefficient relating the relative densities of elastic fibers and age was -0.81 (strong negative correlation), with a high statistical significance (p = 0.0000000002, 95% confidence interval).

**Figure 6:**
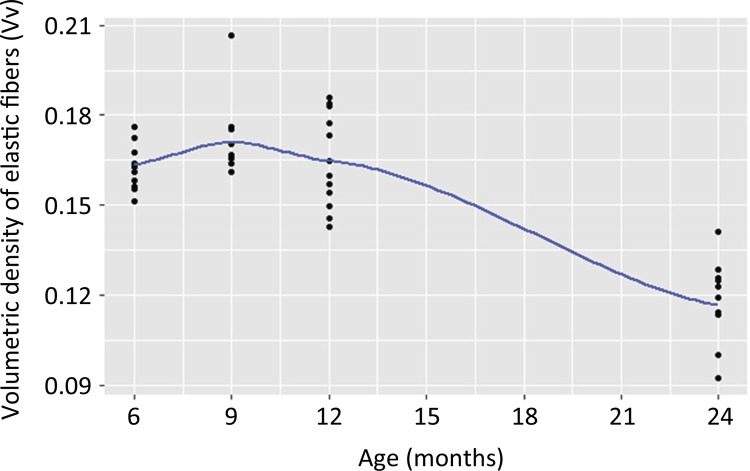
This graphic with trend line shows similar Vv until the age of 12 months and a significant decline when considering rats with 24 months old.

The Vv trend curve in relation to age in the study groups is shown in ( Fig. 6 ). Vv shows stable behavior until 12 months of life, with the greatest reduction observed in G4 (24 months).

**Figure 7:**
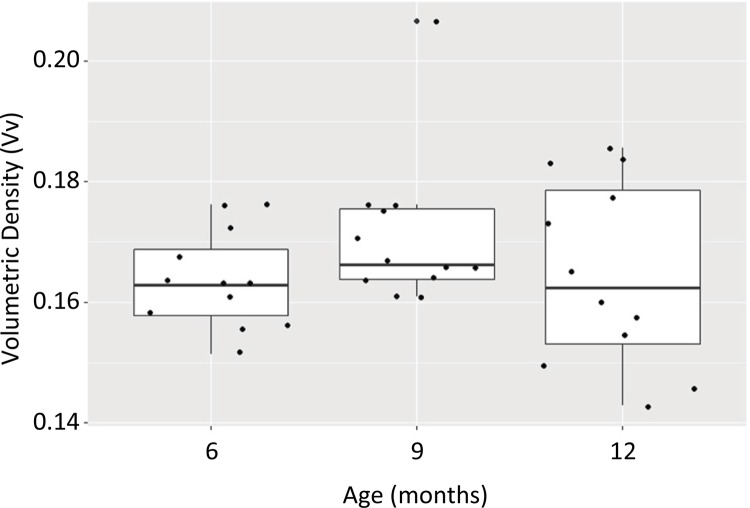
- This figure shows that there is no difference in Vv between the groups with 6, 9 and 12 months old.

The Pearson correlation coefficient for the Vv of elastic fibers of the corpora cavernosa of rats aged ≤12 months was 0.035 (p-value = 0.8). There was no statistical difference between these groups ( Fig. 7 ).

**Figure 8:**
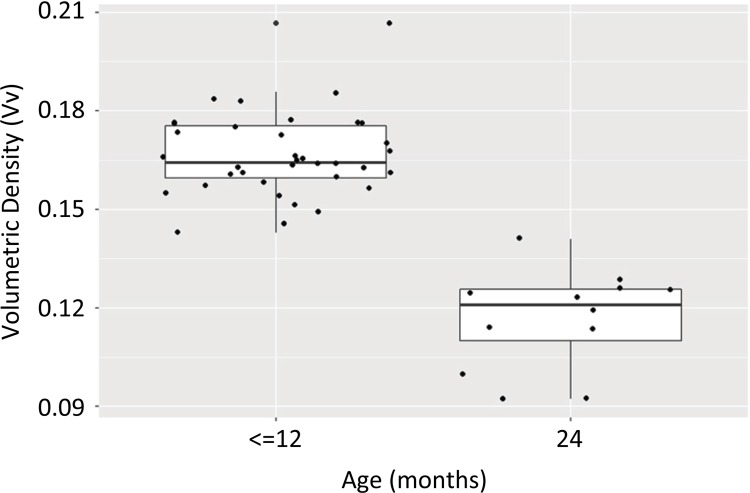
This graph shows the significant difference of Vv of elastic fibers between the group gathering the rats with 6, 9 and 12 months old (Vv:0.1166) and the group of rats with 24 months old (Vv:0.1664) (IC: 0.039-0.059).

Considering the rats aged ≤12 months as a single group, Welch’s *t* -test was used to determine statistical differences between the Vv of elastic fibers in this single group and in G4 (rats euthanized at 24 months). The mean value of Vv in G4 was 0.1166 compared to 0.1664 in the aggregated G1, G2, and G3 group ( Fig. 8 ). The decrease in Vv between the groups was 0.0498 (29.92%), and was statistically significant (confidence interval, CI = 0.039-0.059; p = 0.00000001).

## Discussion

The use of stereology in genitourinary tract studies allows the quantification of its elements, and therefore brings greater objectivity to the analysis. Pinheiro *et al* .^8^ demonstrated the relevance of stereological analysis in determining the relative densities of muscle, collagen, and elastic fibers of the corpus cavernosum of rats at 4 months of age, as well as its effectiveness in describing the behavior of the corpus cavernosum extracellular matrix in pathological states. Also using stereology, Abidu-Figueiredo *et al.* ^9^ proved the effects of diabetes mellitus on the corpus cavernosum of rabbits and showed the reduction of elastic fibers and collagen. In our experience, stereology is a reliable tool, the capacity to provide homogeneous positive test-points for rats at the same age, as well as the capacity to confirm our valid hypothesis in agreement with literature data, base this reliability.

There are considerable differences in the Vv of elastic fibers among mammals with a vascular penis. In humans, the Vv reaches 9%^10^ . In a study analyzing rats aged 4 months, the Vv of elastic fibers was lower than that found in humans, with a mean Vv of 4.9%^8^ . Figueiredo *et al* .^5^ demonstrated the high Vv of elastic fibers in New Zealand rabbits with indices reaching 25.1%. In the present study, the Vv of rats aged ≤12 months was 0.1664 (16.64%), which is greater than that reported in previous studies conducted using rats; there are two technical possibilities to explain such difference: first, computerized counting may underestimate the Vv of fibers depending on the color tone or sensitivity of the software used, and second, manual counting may overestimate Vv, because of the counting of fibers milimetrically distant to the test-point. Another important data is the different ages between our groups, ranging from 6-12 months, and their group of 4 months old.

Few studies have addressed changes in the Vv of elastic fibers of the mammalian corpus cavernosum during the natural aging process. In rabbits, a progressive increase in the Vv of these fibers was observed between 30 and 240 days of life, and stabilization was observed between 240 and 730 days^9^ . In humans and rats, the inverse effect is reported, with reduction of Vv with age^10^ . Shen *et al* .^6^ observed a quantitative reduction in elastic fibers in rats aged 9 to 14 weeks. However, in the present study, there was no significant difference in Vv up to 12 months of age; two factors can explain this difference: first, Shen *et al* .^6^ used a different species of rat, Sprague-Dawley, and second, they studied rats younger than those used in this study.

As the sexual maturity of rats is reached at approximately 50 days, their reproductive senescence varies between 15 and 22 months and, despite these data being collected in females, one can extrapolate these to males^11^ . Thus, our results show that, in adulthood, the Vv of elastic fibers of the corpus cavernosum do not undergo significant changes, while in senescence there is a significant decrease of Vv.

Despite the multifactorial nature of ED, the decrease in the quantity of elastic fibers seems to play an important role by reducing the penile elastic capacity and its firmness during erection. Any degree of loss of these fibers may cause a loss of resistance to distension during erection, with consequent pressure attenuation, culminating in ED^11^ .

The statistical confirmation that elastic fibers of the corpus cavernosum are changed in pathological processes that lead to ED and the direct analysis comparing patients with and without ED clearly demonstrate that these fibers are important. Costa *et al* .^3^ compared biopsies of corpus cavernosum from subjects with and without ED, in individuals with similar ages, and observed the presence of smooth and collagen muscle fibers with similar Vv but with a statistically significant reduction of elastic fibers. In diabetic rabbits, Abidu-Figueiredo *et al* .^9^ observed a reduction in the Vv of elastic fibers of the corpus cavernosum despite the increase in smooth muscle fibers, which indicates that changes in the behavior of elastic fibers seem to be directly associated with the presence of pathological processes resulting in ED. Although it is not the main focus of this study to evaluate the penile functional change with age, the outstanding reduction of elastic fibers Vv in older rats allows us to infer that this reduction may be associated with the ED related to aging.

Aging is an independent risk factor for the development of ED^13^ , and structural changes such as a decrease in the Vv of elastic fibers may therefore play a role in this dysfunction. However, the degree of impairment of erectile function caused by this reduction requires further investigation.

The recognition of the importance of elastic fibers in the physiology of erection, as well as their deterioration with pathological or even natural states such as aging, opens the door for a vast field of study aimed at reversing or preventing damage caused to these fibers. An example of this is the use of low-intensity extracorporeal shock wave therapy and low-intensity pulsed ultrasound (LIPU) which, in studies conducted with animals, has led to changes in the microstructure of the corpus cavernosum. LIPU could even convert the process of penile fibrosis, induced by diabetes mellitus in rats, by increasing the number of smooth muscle fibers and collagen I/III ratio, as well as the amount and shape of elastic fibers^14^ .The present study demonstrated that the Vv of elastic fibers of the corpus cavernosum of rats decreases in senescence. However, further studies are necessary to identify measures that may attenuate this condition.

## Conclusion

The natural aging process in rats was responsible for changes in the volumetric density of elastic fibers of the trabeculae of the corpora cavernosa, as evidenced by a 30% decrease in volumetric density during senescence.

## Footnotes

1 Research performed at Laboratory of Anatomy and Experimental Surgery, Division of Urology, Department of Surgery, Universidade Federal do Paraná (UFPR), Curitiba-PR, Brazil. Part of Master degree thesis, Postgraduate Program in Surgical Clinic. Tutors: Rogério de Fraga and Fernando Lorenzini.
